# Optimization of Ciprofloxacin Adsorption on Clinoptilolite-Based Adsorbents Using Response Surface Methodology

**DOI:** 10.3390/nano13040740

**Published:** 2023-02-15

**Authors:** Barbara Kalebić, Arijeta Bafti, Hrvoje Cajner, Marijan Marciuš, Gordana Matijašić, Lidija Ćurković

**Affiliations:** 1 Faculty of Technology and Metallurgy, University of Belgrade, Karnegijeva 4, 11120 Belgrade, Serbia; 2 Faculty of Mechanical Engineering and Naval Architecture, University of Zagreb, Ivana Lučića 5, 10000 Zagreb, Croatia; 3 Faculty of Chemical Engineering and Technology, University of Zagreb, Marulićev Trg 19, 10000 Zagreb, Croatia; 4 Ruđer Bošković Institute, Bijenička Cesta 54, 10000 Zagreb, Croatia

**Keywords:** ciprofloxacin, clinoptilolite, magnetic nanoparticles, graphene oxide, response surface methodology

## Abstract

The adsorption of the antibiotic ciprofloxacin (CIP) from water solution by natural zeolite–clinoptilolite (CLI), magnetic clinoptilolite (MAG-CLI), and graphene oxide coated magnetic clinoptilolite (GO-MAG-CLI) was investigated. The novel approach of an environmentally friendly and cost-effective microwave-assisted method was applied for the magnetic composite synthesis. Detailed characterization of the prepared composites was achieved. In order to investigate the effect of the initial CIP concentration, pH, temperature, contact time, and type of adsorbent on the adsorption efficiency of CIP, and to obtain the optimal conditions for CIP removal, the response surface methodology central composite factorial design (RSM-CCF) was applied. The results obtained by the RSM-CCF showed that among the studied adsorbents, GO-MAG-CLI had the highest adsorption capacity for CIP, achieved for the initial concentration of 48.47 mg dm^−3^ at a pH of 5 and 24.78 °C after 19.20 min of contact time. The adsorption kinetics studied for the initial CIP concentration range of 15–50 mg dm^−3^ followed Lagergren’s pseudo-second-order model, and the Langmuir isotherm was the most suitable one to describe the CIP adsorption onto GO-MAG-CLI.

## 1. Introduction

Since Flaming’s discovery in 1928, antibiotics have been widely used as a pharmaceutical agent in both human and veterinary medicine, but also as a growth promotor in livestock [1]. Although they have saved a million lives, the uncontrolled production and consumption of antibiotics is becoming one of the major environmental issues nowadays, with already noticeable consequences for human health. Only a small portion of consumed antibiotic substances are adsorbed in human or animal bodies, with most of the substances being discharged unmetabolized into natural waters through untreated sewage and wastewater streams [2,3]. Elevated concentrations of antibiotics in the environment, in addition to a toxicological effect on non-target organisms, lead to the development of antibiotic-resistant bacteria, which is considered one of the biggest threats to global health today [4].

Ciprofloxacin (CIP) is one of the widely applied second-generation fluoroquinolone antibiotics. It has the most potent effect against Gram-negative bacilli bacteria but is also effective against some Gram-positive ones [5]. Due to the extensive use of fluoroquinolone antibiotics, the detected concentrations of CIP in surface waters range from ng to mg dm^−3^ [6]. The main characteristic of the CIP molecule is its zwitterionic nature, with two p*K*a values at 5.90 ± 0.15 and 8.89 ± 0.11 for the carboxyl and amine groups, respectively [7,8]. This strong pH dependence of the CIP molecule’s charge influences its removal from water media.

Among different methods that have been investigated and used in water treatment, adsorption has proved to be a promising one for the removal of antibiotics, since it is a relatively simple and inexpensive technique that is insensitive to the antibiotics’ toxicity [9]. A keen interest in the field of adsorption exists for improved and advanced low-cost adsorbents with large surface areas and high adsorption capacities. Therefore, various adsorbents have been investigated for the removal of CIP from water, such as metal oxide nanoparticles [10,11], carbon-based adsorbents [12,13], and naturally occurring minerals [14,15,16].

Natural zeolites stand out among the adsorbents due to their unique structural properties, thermal stability, and availability, which make them low-cost materials. Clinoptilolite (CLI) is the most abundant natural zeolite and is widely investigated in the field of water treatment [17]. The adsorption capacity of CLI for various types of pollutants can be ascribed not only to its unique structure but also to its tendency to be modified without significant structural changes. The modification of the CLI surface with different metal oxide nanoparticles could enhance its adsorption capacity for the specific types of pollutants by enlarging its active surface area [18,19,20]. In recent years, the synthesis and utilization of magnetic iron oxide nanoparticles such as magnetite (Fe_3_O_4_) and maghemite (*γ*-Fe_2_O_3_) in the removal of different inorganic and organic compounds from water media have been widely studied due to the nanoparticles’ nano-size, high surface area-to-volume ratio, and superparamagnetic properties [21,22,23]. The magnetic iron oxide nanoparticles themselves have been shown to be efficient in adsorbing CIP from water by forming a bridging-bidentate surface complex with iron oxide nanoparticles [6,11]. Thus, the coating of CLI with magnetic nanoparticles (MAG-CLI) proved to be effective in the removal of metal cations [24,25] as well as pharmaceutical compounds [26] from water solutions. The removal efficiency of the antibiotic cephalexin from water onto CLI is increased by 65% after CLI coating with Fe_3_O_4_ nanoparticles [27], while the coating of natural zeolite with *γ*-Fe_2_O_3_ nanoparticles leads to high adsorption potential for different pharmaceutical compounds, with a removal efficiency of more than 95% within 10 min of adsorption [26]. In addition to the large active surface area, low toxicity, and simple synthesis method, iron oxide nanoparticles (MAG) possess magnetic properties that can induce magnetism to CLI and facilitate its separation from the liquid phase after the adsorption by applying the external magnetic field [21,28]. This could overcome the common adsorption problem of adsorbent separation.

Another candidate for the improvement of CLI adsorption properties is graphene oxide (GO)—a single monomolecular layer of graphite with various oxygen-containing functionalities, such as epoxy, carboxyl, carbonyl, and hydroxyl groups [29]. The main characteristic of GO layers is the large specific surface area of around 2400 m^2^ g^−1^ [30], which enables GO to be used as an efficient adsorbent for different organic pollutants, such as dyes [31], aromatic compounds [32], and antibiotics [33]. The loading of GO onto CLI leads to their bonding via electrostatic and hydrophobic interactions and hydrogen bonds, which can enlarge CLI’s surface area by ten times [29]. According to Chen et al., pure GO exhibits high adsorption capacity for CIP for the initial concentration range of 1–20 mg dm^−3^, and the adsorption was mainly controlled by the electrostatic attractions and H-bonding interactions [34]. Furthermore, the synergistic effect of magnetic nanoparticles and GO proved to be efficient in CIP removal through electrostatic and *π*–*π* electron interactions [35].

To model the adsorption process and to determine the optimal parameters within given criteria, the statistical approach of the response surface methodology (RSM) is commonly used. The basic purpose of the RSM is to model the process and optimize the parameters by lowering the number of experiments that need to be performed. The RSM has the potential to determine the effects of multiple factors and their interactions on one or more responses using fewer tests, while also obtaining higher accuracy [10,36]. The most common design used in the RSM is the central composite design (CCD), which is an extended factorial design with the ability for quadratic model evaluation [37].

This study presents a novel route for preparing a novel composite consisting of magnetic nanoparticles and GO particles in a clinoptilolite matrix (GO-MAG-CLI). For the route, we employed a relatively simple and cost-effective method of microwave-assisted synthesis in combination with ultrasonication. The coating of both magnetic iron oxide nanoparticles and graphene oxide onto CLI should improve CLI’s adsorption capacity, as well as facilitating composite separation from the liquid phase. The adsorption performances of GO-MAG-CLI were compared with those of pristine CLI and magnetic CLI (MAG-CLI) for CIP removal from aqueous solutions. The optimum operating conditions for the adsorption process providing the maximum adsorption capacity for CIP were determined using the CCD based on the RSM. Additionally, the detailed characterization of the prepared composites was performed, and the kinetic and isotherm studies were carried out to investigate the CIP adsorption mechanism.

## 2. Materials and Methods

### 2.1. Materials

The clinoptilolite-rich zeolitic tuff obtained from the Serbian deposit Slanci was used as a starting material in this study. The quantitative powder X-ray diffraction (PXRD) analysis and Rietveld refinement calculations [38] were performed to confirm the mineral identity of the zeolitic tuff. The zeolitic tuff was stated to be of >80 wt.% purity on clinoptilolite (CLI) with quartz (<7.5 wt.%) and feldspar (<13 wt.%) as the major satellite phases. The cation exchange capacity (CEC) of the CLI, which was measured using a standard procedure [39], was 162.2 mmol M ^+^/100 g.

Prior to the composites’ synthesis and adsorption experiments, the CLI sample was sieved, washed with deionized water to remove impurities, and then dried in an oven at 105 °C overnight to a constant mass. The particle size range of 0.063–0.125 mm was used in the experiments, since previous studies showed the best adsorptive performance in that particle size range [40,41].

For the MAG-CLI preparation, iron (III) chloride hexahydrate (FeCl_3_·6H_2_O, 99%, AnalaR NORMAPUR^®^ ACS, VWR Chemicals, Darmstadt, Germany) and iron (II) sulfate heptahydrate (FeSO_4_·7H_2_O, 98%, Alfa Aesar, Kandel, Germany) were used. A commercial graphene oxide water dispersion (4 mg cm^−3^, Graphenea, San Sepastián, Spain) was used for the coating of MAG-CLI. The studied pharmaceutical compound ciprofloxacin (CIP, 98%, Acros Organics, Waltham, MA, USA) was used as received.

A stock solution of CIP (50 mg dm^−3^) was prepared by dissolving a required amount of CIP in deionized water, which was kept at a temperature of around 4 °C. The stock solution was diluted with deionized water to obtain the desired concentrations (15–50 mg dm^−3^) used in the adsorption experiments.

All used chemicals were of analytical grade, and all experiments were carried out under controlled conditions. The adsorption experiments were performed in a thermostated laboratory incubator–shaker, where the temperature was kept constant to within ±0.7 °C; the samples were weighed to a four-digit accuracy, and the solution concentrations were also determined to a four-digit accuracy. Deionized water was used in all experiments.

### 2.2. Microwave-Assisted Synthesis of Magnetic Nanoparticles (MAG)

The magnetic nanoparticles (MAG) were prepared according to the already reported literature procedure [42]. Aqueous solutions of two iron salts—FeCl_3_·6H_2_O (0.4 M) and FeSO_4_·7H_2_O (0.2 M)—in a molar ratio of 2:1 were mixed properly. Subsequently, the water solution of NaOH (2 M) was added to the iron salt solution, and the solution was microwave (MW)-irradiated (Microwave Reaction System SOLV, Multiwave PRO, Anton-Paar GmbH, Graz, Austria). The obtained black precipitate (MAG) was separated from the liquid phase, washed several times with deionized water, and dried until reaching a constant mass.

### 2.3. Microwave-Assisted Synthesis of Magnetic Clinoptilolite (MAG-CLI)

The magnetic clinoptilolite (MAG-CLI) was prepared following the slightly modified procedure described by Iskandar et al. [43]. Briefly, the aqueous solutions of FeCl_3_·6H_2_O (0.4 M) and FeSO_4_·7H_2_O (0.2 M) in the molar ratio of 1:2 were mixed properly with the CLI water suspension at room temperature. Subsequently, the aqueous solution of NaOH (2 M) was added dropwise to the prepared suspension until the pH reached 10. The formed black suspension was transferred to four Teflon vessels and MW-irradiated (Microwave Reaction System SOLV, Multiwave PRO, Anton-Paar GmbH, Graz, Austria) at 200 °C for 5 min under high stirring. The inner pressure and temperature were monitored during the synthesis process. The synthesized dark brown precipitate after the MW-assisted co-precipitation reaction was separated from the suspension by centrifugation and washed several times with deionized water until showing a negative reaction to the chloride ions. Finally, the obtained MAG-CLI was dried in the oven at 100 °C until reaching a constant mass. The sample was left to cool to room temperature before its further use.

### 2.4. Coating of MAG-CLI with Graphene Oxide (GO-MAG-CLI)

A graphene oxide (GO) water dispersion (3.6 cm^3^) and 11.4 cm^3^ of ethanol were mixed and ultrasonicated for 30 min for the expanded interlayer GO structure exfoliation [44]. Then, the MAG-CLI powder was added to the GO dispersion at a GO/MAG-CLI ratio (*w/w*) of 1:2. The suspension was subsequently ultrasonicated (Giorgio Bormac, DU-32) for 30 min (40 kHz, 120 W), stirred for 30 min several times, and then placed in an oven at 80 °C overnight.

### 2.5. Adsorbents’ Characterization

An analysis of the mineral phases present in the samples was performed using the powder X-ray diffraction method (PXRD). The PXRD patterns were obtained using a D8 Advance (Bruker, Billerica, MA, USA) X-ray diffractometer with Cu*K*α radiation with an acceleration of 40 kV and a 25 mA current in the Bragg–Brentano focusing geometry. The analysis was conducted in step-scan mode with a 0.02° 2*θ* step, in the 2*θ* range of 5–65°, at 0.6 s per step. The quantitative analysis of the obtained PXRD diffractograms was performed using the Crystal Impact Match! software package.

To gain insight into the nanoparticles’ morphology, a thermal field-emission scanning electron microscope (FE-SEM) (JSM-7000F, Jeol Ltd., Tokyo, Japan) was used.

Room temperature Mössbauer spectra were recorded using a classic transmission spectrometer using a WissEl configuration (Wissenschaftliche Elektronik GmbH, Starnberg, Germany) with a ^57^Co-Rh gamma Mössbauer source. The recorded spectra were relatively calibrated to the *α*-Fe reference sample, and the obtained data were processed using the MossWinn 4.0 program.

Raman measurements were performed via confocal micro-Raman spectroscopy using a Horiba Jobin Yvone T64000 (Kyoto, Japan) equipped with a solid-state laser with a wavelength of 532.5 nm and a 50× magnification large working distance objective in the range of 300–3500 cm^−1^.

The BET surface area and porosity characteristics were determined from the nitrogen adsorption–desorption isotherms at –196 °C using an ASAP 2000 apparatus (Micromeritics Corporation, Norcross, GA, USA). Prior to the analysis, the samples were degassed at 150 °C for 10 h. The specific surface area was calculated using the Brunauer–Emmett–Teller (BET) method, while the total pore volume (*V*_tot_) was determined from the desorption isotherm at *p*/*p*_0_ = 0.998. The pore size distribution of the sample was determined by the Barret–Joyner–Halenda (BJH) model from the data of the adsorption branch of the nitrogen isotherms.

X-ray photoelectron spectroscopy (XPS) measurements were carried out on the SPECS system under the UHV conditions, with the typical chamber pressure in the range of 10 to 7 Pa. The XPS instrument was equipped with the Phoibos MCD 100 electron analyzer and the monochromatized X-ray source of 1486.74 eV (Al *Kα* line). The survey XPS spectra were recorded with a pass energy of 50 eV, while the pass energy of 20 eV was used for the photoemission measurements around the atomic core levels (O 1s, C 1s, Si 2p, Al 2p, Ca 2p, K 2p, Fe 2p). All spectra were calibrated according to the C 1s peak, placed at the binding energy of 285.0 eV.

The thermal behavior of the samples was analyzed using the simultaneous differential thermal analysis and thermos-gravimetric analysis (DTA/TGA) apparatus Netzsch STA 409C (Selb, Germany). The samples were heated from room temperature to 800 °C at a heating rate of 10 °C min^−1^ in synthetic air with a flow rate of 30 cm^3^ min^−1^, while corundum was used as a reference.

The zeta potentials were measured using a Zetasizer Ultra (Malvern Panalytical, Malvern, UK). In short, 0.1 g of sample was suspended in 100 cm^3^ of deionized water and ultrasonicated for 20 min. The pH of the suspension was then adjusted in the range of 2 to 12 using HCl or NaOH (0.1 mol dm^−3^). Before each measurement, the suspensions were ultrasonically treated for 30 s. The suspensions’ pH values were adjusted using a Mettler Toledo (Columbus, OH, USA) digital pH meter.

The magnetic properties were measured using a LakeShore 8607 Series VSM (vibrating sample magnetometer; Lake Shore cryotronics, Westerville, OH, USA) at room temperature and with an applied magnetic field strength of 2 T.

### 2.6. CIP Adsorption Experiments

To study the CIP adsorption onto the CLI-based adsorbents, the batch method using 0.2 g of the adsorbent in 50 cm^3^ of CIP solution was used. The effect of the initial CIP concentration (15–50 mg dm^−3^), pH (5–9), temperature (10–25 °C), time (5–20 min), and adsorbent (CLI, MAG-CLI, GO-MAG-CLI) on the adsorption efficiency was investigated. The pH adjustment was performed using HCl or NaOH solutions (0.1 mol dm^−3^). The suspensions were shaken in a thermostatic orbital shaker–incubator (ES-20/80, Biosan, Riga, Latvia) at a rotation speed of 170 rpm for 5 to 60 min. The solid was separated from the suspension by vacuum filtration and then additionally filtered through a 0.22 μm nylon filter. The CIP concentration was measured in the filtrate using a standard procedure with a UV-Vis spectrometer (Spekol 2000, Analytic Jena, Jena, Germany) at 278 nm [45]. The amount of CIP adsorbed onto the adsorbent after time *t* (*q*_t_) was calculated using the following formula:

*q*_t_ = (*C*_0_ − *C*_t_)/*m* × *V* (1)

where *C*_0_ (mg dm^−3^) is the initial CIP concentration, *C*_t_ (mg dm^−3^) is the CIP concentration after time *t*, *V* (dm^3^) is the volume of the CIP solution, and *m* (g) is the adsorbent mass.

### 2.7. Design of Experiment

The optimization of the CIP adsorption process onto CLI-based adsorbents was conducted using the Design Expert software (version 13). The purpose of the design and analysis of the experiment (DoE) is to obtain an empirical mathematical model, which will predict the outcome of a dependent variable apropos a group of independent variables. Furthermore, by applying the DoE, the significance of each independent variable and its combination with the outcome can be quantified.

The results of the adsorption experiments were obtained using a face-centered central composite design (CCF) with five independent variables (factors) varied across three levels (Table 1). The chosen response variable was the adsorbents’ capacity for CIP.

The number of experiments performed for the full factorial CCF design was calculated according to the equation *N* = 2*^k^* + 2*k* + *n*_c_, where *k* stands for the number of factors, 2*^k^* represents the number of factorial points, 2*k* is the axial points, while *n*_c_ refers to the number of replicates at the center point.

A statistical analysis including an analysis of variance (ANOVA) and the prediction of regression coefficients, response surface, and contour plots was employed. The level of significance was 5%, which is a commonly used threshold to classify the statistical significance of the evaluated statistical properties of the model.

## 3. Results and Discussion

### 3.1. Characterization of Synthetized Adsorbents

**Figure 1:**
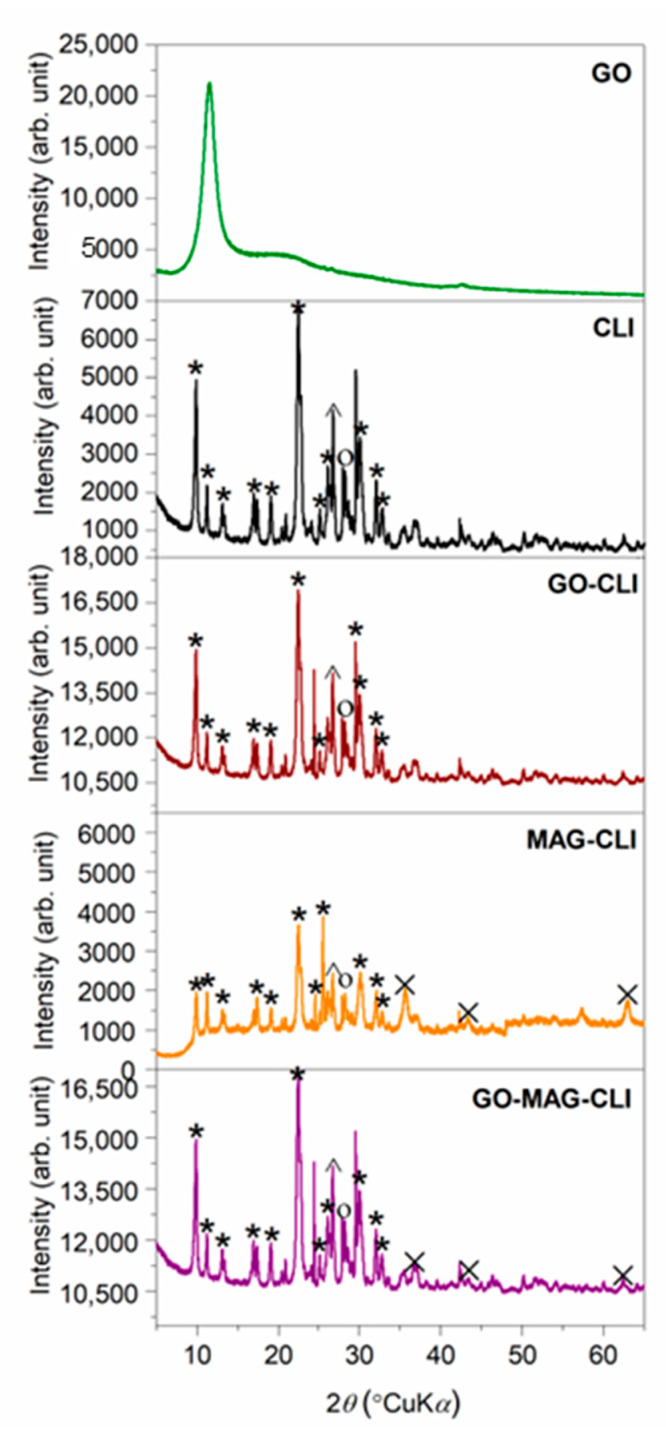
PXRD patterns of CLI, MAG-CLI, GO-MAG-CLI, GO-CLI, and GO (*—clinoptilolite; ˄—quartz; ○—feldspar; ×—maghemite).

To investigate the changes in CLI crystallinity after modifications, the PXRD analysis was performed (Figure 1). All diffractograms related to CLI-based composites displayed the peaks characteristic for the clinoptilolite lattice, which was identified by card no. 01-080-1557 from the PDF crystallographic base. The CLI crystal cell unit was found to be monoclinic with C2/m space groups. In the diffractogram of MAG-CLI, new peaks appeared indicating the presence of iron oxide particles (card no. 00-039-1346) with a cubic crystal cell unit [21,24]. Since Fe_3_O_4_ and *γ*-Fe_2_O_3_ have similar cubic spinel crystal structures and almost identical lattice parameters, it is difficult to distinguish them from one another based only on the PXRD peak positions. The quantitative analysis of the obtained XRD peaks of pure MAG and MAG-CLI was performed using the reference intensity ratio (RIR) method (Figures S1 and S2). The obtained results (Table 2) indicated that the synthesized magnetic particles contain a handful of various iron oxide phases. In the pure MAG particles, the content of Fe_3_O_4_ is only 10 wt.%, while *γ*-Fe_2_O_3_ presents the dominant phase in the sample. Additionally, as an iron oxide–hydroxide, goethite could be formed as a satellite phase in the MW-assisted co-precipitation of Fe_3_O_4_. It is weakly magnetic, so its presence in an MAG sample could affect the MAG’s magnetic properties [46]. On the other hand, the MW-assisted synthesis of magnetic CLI resulted in the precipitation of only *γ*-Fe_2_O_3_ on the CLI surface. Although the obtained result indicates the presence of *γ*-Fe_2_O_3_ in the MAG-CLI composite, it should be noted that there is a possibility of previous formation of Fe_3_O_4_, which then gradually converted to *γ*-Fe_2_O_3_ after exposure to air [23].

In the pattern of GO, a high-intensity diffraction peak was observed at 2*θ* = 11.50°, which is characteristic of GO layers [47].

**Figure 2:**
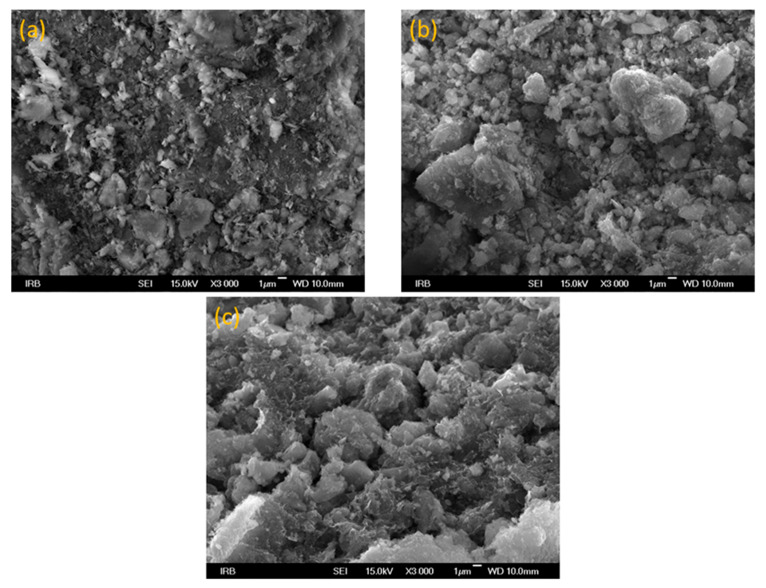
SEM images of (**a**) CLI, (**b**) MAG-CLI, and (**c**) GO-MAG-CLI.

To investigate the surface morphologies of the prepared adsorbents and to determine the particle size, the FE-SEM analysis was performed. Figure 2 shows the SEM images of CLI (Figure 2a), MAG-CLI (Figure 2b), and GO-MAG-CLI (Figure 2c). As can be seen in Figure 2b, the formation of MAG particles (particle sizes estimated in the range of 20–40 nm) on the CLI surface did not affect the CLI structure. Additionally, the agglomeration of spherical magnetic nanoparticles due to their formation by co-precipitation on the CLI surface was observed. This behavior can be assigned to the high surface energy of the smaller-sized particles, which attract other particles to form particle agglomerates. The same finding was reported for the synthesis of Fe_3_O_4_-coated clinoptilolite using the standard co-precipitation method [19]. The addition of GO (Figure 2c) did not have a significant effect on the CLI’s surface morphology. The large GO sheets on GO-MAG-CLI were not evident due to the low GO loading.

**Figure 3:**
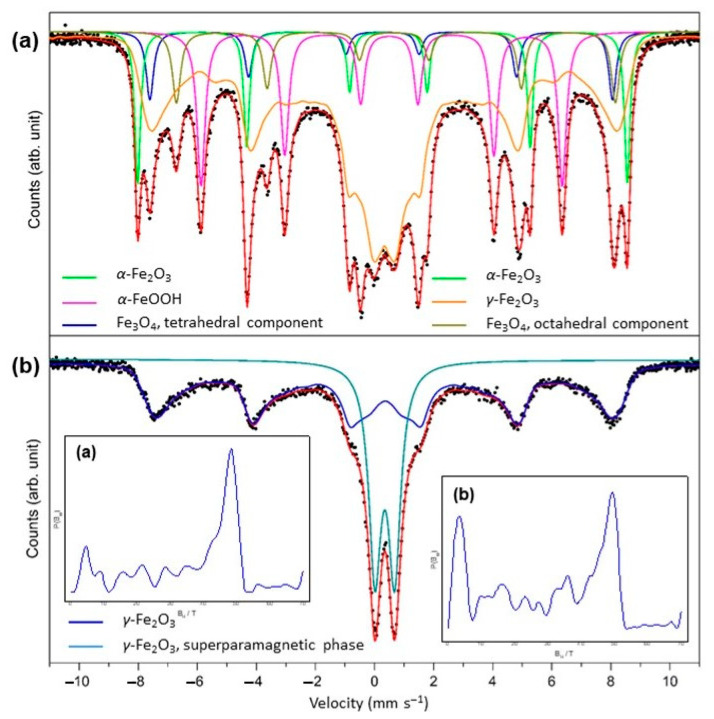
^57^Fe Mössbauer spectra of (**a**) MAG and (**b**) MAG-CLI samples (•–experimental data). The insets show the distribution of *B*_hf_.

To evaluate the magnetic iron oxide phases in MAG and MAG-CLI samples, Mössbauer spectroscopy was performed. The ^57^Fe Mössbauer spectra of pure MAG and MAG-CLI samples are shown in Figure 3. The calculated Mössbauer parameters and phase identification results are summarized in Table 3 and Table 4.

The Mössbauer spectrum of magnetic iron oxide nanoparticles depends on the structural properties resulting from the synthesis method and the particle size. As was already determined by the PXRD analysis, the dominant iron oxide phase in MAG and MAG-CLI is *γ*-Fe_2_O_3_. The spectrum of MAG (Figure 3a) was fitted as a superposition of four sextets. The prominent central doublet in the spectrum of MAG nanoparticles is a consequence of the fast superparamagnetic relaxation of the smaller crystal domains of the *γ*-Fe_2_O_3_ phase in the sample. Furthermore, a broad sextet pattern described by the distribution of the hyperfine field, *B*_hf_, can be attributed to the broad range of crystal domains sizes of the *γ*-Fe_2_O_3_ phase, above the superparamagnetic relaxation threshold [48].

The formation of *γ*-Fe_2_O_3_ usually accompanied the formation of Fe_3_O_4_ due to the slow magnetite oxidation by oxygen in the air [49], which results in the formation of the *γ*-Fe_2_O_3_ layer on the material’s surface. This impact becomes significant in nanoscale materials due to the large surface area, where it can be observed even in a single crystal. Moreover, the starting reaction mixture and the mechanism of the present MW-assisted synthesis allow the formation of a multiphase iron oxide material [50]. A sequence of several mechanisms can be explained by the fast nucleation in an aqueous system, the sudden formation of particles in the reaction mixture, as well as the very short synthesis time.

However, the coating of iron oxide nanoparticles on CLI resulted in the formation of only maghemite in two magnetic states. The primary nominal component of the MAG-CLI spectrum corresponds to a sextet, which indicates that these particles are larger than 20 nm and magnetic, while smaller particles in the superparamagnetic state below 10 nm correspond to a spectral doublet. According to the previous studies on the influence of ultrasound irradiation on the physicochemical properties of iron oxide particles [51,52], the applied sonication conditions should not affect the magnetic properties of iron oxide particles. Thus, it was assumed that the iron oxide phases present in MAG-CLI remained unchanged after the GO coating.

**Figure 4:**
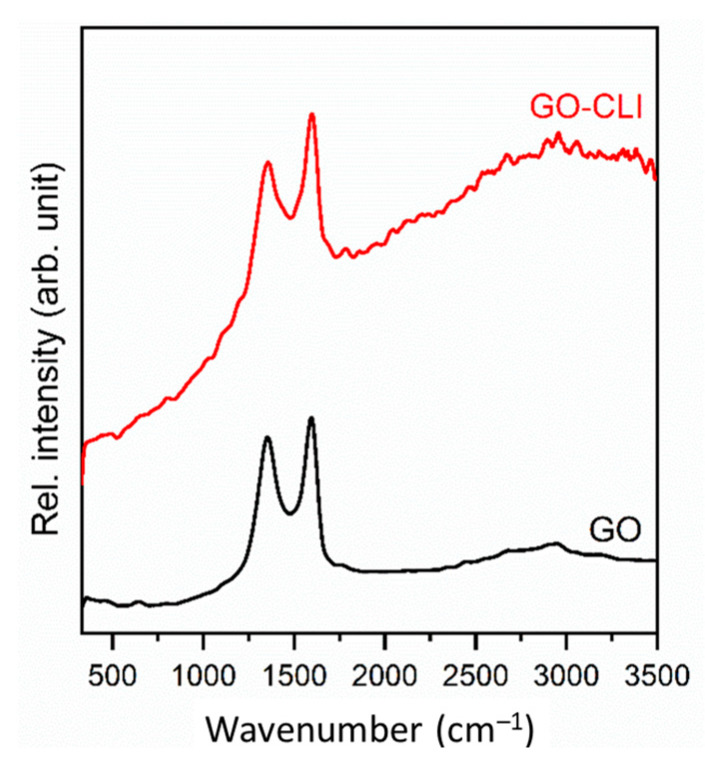
Raman spectra of GO and GO-CLI.

Raman spectroscopy based on band intensity and band surface measurements was applied for the GO detection in the prepared composite. The Raman spectra of the used commercial GO and GO-coated clinoptilolite (GO-CLI) are shown in Figure 4. The characteristic bands of the GO spectra at around 1350 cm^−1^ (band D) and 1600 cm^−1^ (band G) are the results of the sp^3^ structural disorder of the carbon atom and sp^2^ vibrations of the carbon atoms present in the hexagonal graphite structure, respectively [53]. These peaks, with an unchanged intensity, are also evident in the GO-CLI spectrum, which indicates the presence of GO in the CLI structure.

The specific surface area (*S*_BET_) and total pore volume (*V*_tot_) of each adsorbent are summarized in Table 5. The *S*_BET_ of CLI increased with its transformations. Compared to the pristine CLI, the addition of iron oxide doubled the CLI surface area due to the increase in a number of active sites available for adsorption. Similar results have already been obtained for a natural zeolite coating with magnetite nanoparticles [19,23]. On the other hand, the increase in the CLI-specific surface area after the GO coating was lower than after coating it with iron oxide, but in accordance with the literature data previously reported for the GO nanosheets grafted to natural CLI [54]. Furthermore, the synergetic effect of both iron oxide nanoparticles and GO led to an increase in CLI *S*_BET_ three times. This increase could be ascribed to the large surface area of the coated materials (MAG~80 m^2^ g^−1^; GO~400 m^2^ g^−1^) [54,55]. The same trend was observed for the *V*_tot_.

**Figure 5:**
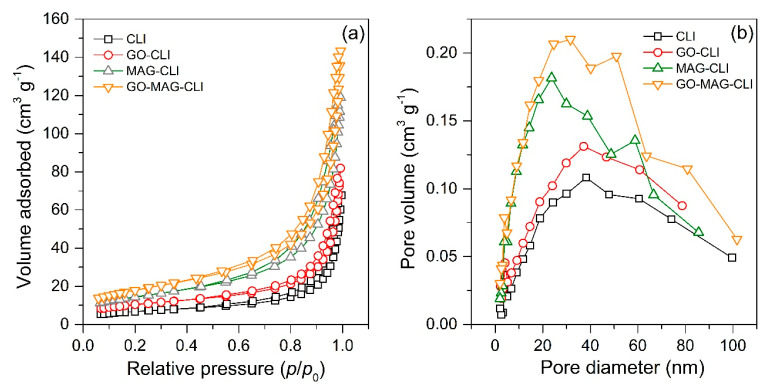
(**a**) Nitrogen adsorption/desorption isotherms and (**b**) pore size distributions.

The N_2_ adsorption/desorption isotherms and pore size distribution (PSD) are given in Figure 5. Based on the IUPAC classification, all samples show type IV adsorption/desorption isotherms, which is typical for clinoptilolite’s structure with both micropores (as a result of the lattice’s structural features) and mesopores formed by the zeolite modification (Figure 5a). The presence of a type H3 hysteresis loop in the *p*/*p*_0_ range of 0.5–1 for all samples indicates that the zeolitic tuffs are rich in clinoptilolite and can be ascribed to multilayer adsorption and capillary condensation, either in mesopores of impurities (feldspar, quartz, etc.) or in the space between zeolite crystallites [56]. Moreover, the PSD of the samples differ mutually (Figure 5b). The differences can be explained by the formation of an additional secondary pore structure during the formation of the iron oxide nanoparticles and GO coating at the clinoptilolite surface [57].

**Figure 6:**
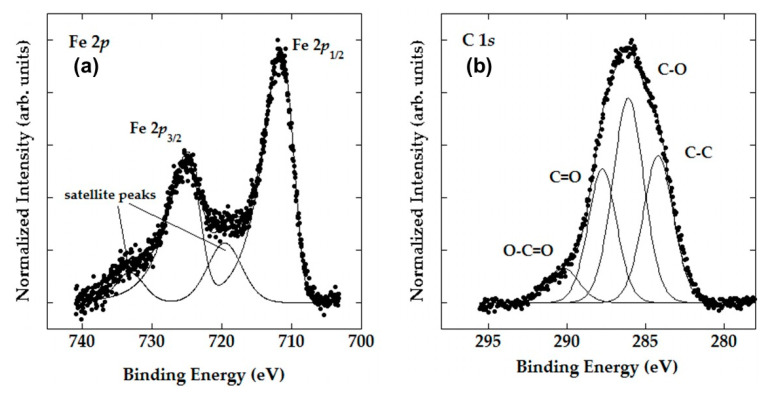
High-resolution XPS spectra of the (**a**) Fe 2p region of MAG-CLI and (**b**) C 1s region of GO-MAG-CLI samples.

To gain a better insight into the surface properties of the prepared CLI-based adsorbents, an XPS analysis was performed. The high-resolution spectra of the Fe 2p and C 1s regions obtained using XPS measurements for MAG-CLI and GO-MAG-CLI, respectively, are shown in Figure 6. The results indicate that the iron is present in the Fe^3+^ oxidative state [35,58]. This is in accordance with the obtained results for the Mössbauer spectroscopy. The deconvoluted C 1s peaks in Figure 6b show peak binding energies of 284.8, 286.2, 288.2, and 290.1 eV, which correspond to C–C, C–O, C=O, and O–C=O bonds, respectively, indicating the presence of oxidized graphene sheets [59].

Table 6 gives the relative elemental composition of CLI and the synthesized composites, which was determined based on the studied elements’ XPS peak intensities. During the CLI’s modification with MAG, the content of exchangeable cations (K and Ca) negligibly decreased while the content of Fe increased. The Fe content increase was significantly higher than the decrease in exchangeable cations content, so it can be concluded that the conversion of CLI to MAG-CLI occurs not only through an ion exchange reaction but also through Fe species precipitation on the CLI surface. The carbon present in the CLI and MAG-CLI spectra (Figure S3) came from the contamination layer caused by sample handling in the air. The binding energy detected in the carbon C 1s XPS spectra of CLI was 284.7 eV, which can be assigned to C–C and C–H bonds [60].

**Figure 7:**
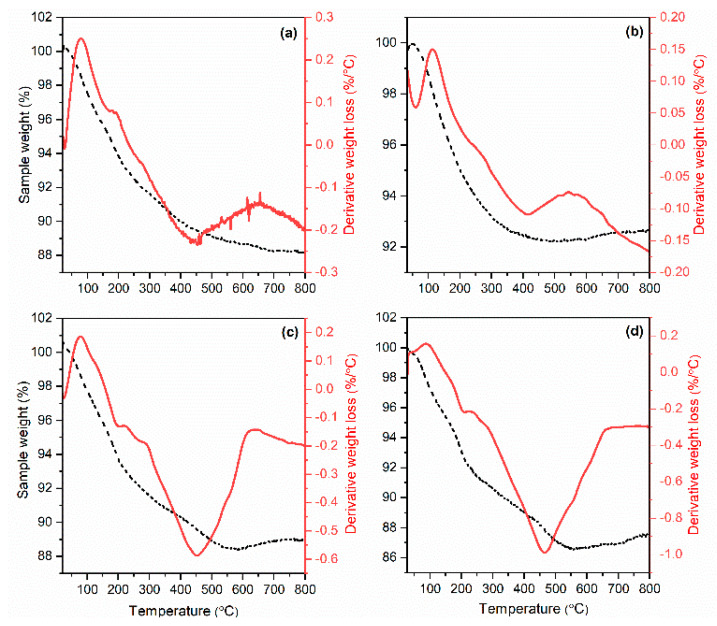
TG (dash) and DTG (solid) curves of (**a**) CLI, (**b**) MAG-CLI, (**c**) GO-MAG-CLI, and (**d**) GO-CLI.

The thermograms of CLI, MAG-CLI, and GO-MAG-CLI (Figure 7) show rapid weight loss (5–10 wt.%) in the temperature range from 25 up to 300 °C, which could be attributed to the loss of the water located mostly in the CLI cavities [61]. Generally, the endothermic peak in that range is produced by the water molecule adsorption phenomenon from the different cationic sites [62]. In the samples where GO is present, the first decomposition of unstable oxygen groups occurs at lower temperatures (below 200 °C), which overlap with the water loss. Afterwards, at around 200 °C, a small exothermic peak can be observed, which corresponds to the decomposition caused by the elimination of more stable oxygen functional groups. The third step of the weight loss occurs in a temperature range from 400 to 500 °C, which can be attributed to the combustion of the carbon skeleton of the GO [63,64]. The total weight loss results for all analyzed samples are similar, varying from 8 to 12 wt.%.

**Figure 8:**
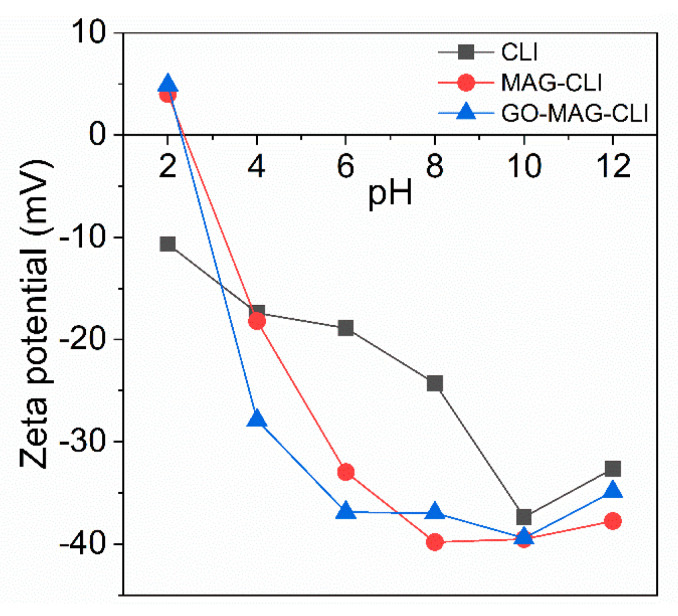
Zeta potential as a function of pH for CLI, MAG-CLI, and GO-MAG-CLI.

The graphs of the zeta potential as a function of pH (Figure 8) show that the zeta potential of CLI changed with modifications, although all three samples had a negatively charged surface across a wide range of pH values from 3 to 12. For MAG-CLI and GO-MAG-CLI, the point of zero charge (PZC) was at a pH = 2.4. The surface charge of GO-MAG-CLI was positive at a pH below PZC with the –OH and –COOH groups of GO in the form of –OH^2+^ and –COOH^2+^. At pH values higher than PZC, these groups were ionized and the GO-MAG-CLI surface was negatively charged. Due to the CIP zwitterionic nature, its adsorption on CLI-based composites is strongly pH-dependent. Thus, the GO-MAG-CLI has the highest adsorption ability for CIP in slightly acidic conditions (pH~5) due to the electrostatic attraction between the negatively charged adsorbent’s surface and the cationic form of the CIP molecule.

**Figure 9:**
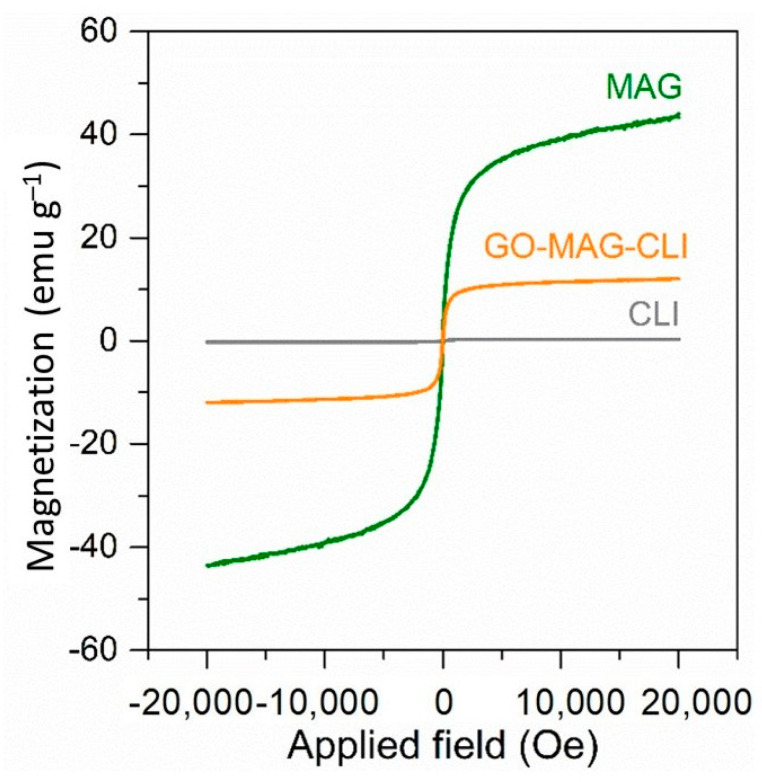
The magnetization curves of MAG, CLI, and GO-MAG-CLI.

Figure 9 presents the magnetization curves of GO-MAG-CLI, pure CLI, and iron oxide magnetic nanoparticles (MAG) as reference materials. Both the MAG and GO-MAG-CLI showed similar superparamagnetic behaviors, with saturation magnetization values of 44.07 and 12.00 emu g^−1^ within a magnetic field lower than 2 T, respectively. The lower saturation magnetization value of GO-MAG-CLI was the result of MAG precipitation on the CLI surface. However, the obtained value indicates that the precipitation of iron oxide nanoparticles onto CLI can induce magnetism to CLI, since CLI itself does not exhibit magnetic properties. Additionally, GO-MAG-CLI retains magnetic properties after the CIP adsorption (not shown), suggesting that the magnetic separation of the spent GO-MAG-CLI from the water media is possible.

### 3.2. Optimization of CIP Adsorption Process Using Model

Based on the RSM–CCF design, 90 experiments were performed (12 axial points, 48 factorial or cube points, and 30 replicates at the center point) to model the adsorption of CIP onto CLI, MAG-CLI, and GO-MAG-CLI and to evaluate the optimum adsorption parameters.

**Figure 10:**
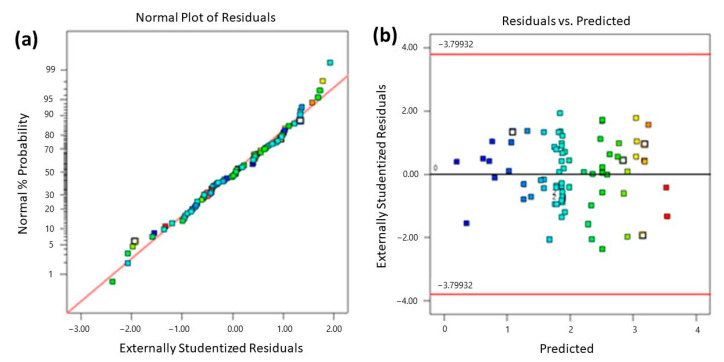
(**a**) A normal probability plot of residuals and (**b**) studentized residuals vs. predicted values plot.

Firstly, the data were studied to verify the normality of the residuals via the normal probability plot of the residuals (Figure 10a). As observed in Figure 10a, the residuals show normality, which indicates the adequacy of the predicted model. The plot of the internally studentized residuals against the predicted response values (Figure 10b) shows the even scattering of points above and below the x-axis. Regarding the previously mentioned facts, it can be concluded that the fitted model is adequate, with a high coefficient of determination (*R*^2^ = 0.9989).

An assessment of the validity and adequacy of the model and the most important effects and probable interactions between the variables was performed using an ANOVA—a statistical method that allows the hypotheses to be tested based on the model parameters [10,65]. The ANOVA results for the polynomial model are outlined in Table S1. Based on the table, the *F*- and *p*-values of the developed model are equal to 596.73 and <0.0001, respectively. Therefore, the polynomial model is highly significant for CIP removal by CLI-based adsorbents (Table S1). According to the results, the variables with the greatest impact on the CIP adsorption are the linear parameters of the initial CIP concentration (A); pH value (B); type of adsorbent (E); and interaction terms BE, CE, and BCE.

To describe the process of CIP adsorption onto the CLI and CLI-based adsorbents, higher-order polynomial models were developed by introducing the adsorption results, i.e., the adsorption capacities. In order to achieve the best statistical properties of the model, the response had to be transformed by applying a square root transformation. The relative impact of the factors on the CIP removal can be identified by analyzing the values of the coefficients of the final equation, which is expressed in terms of coded factors using Equation (2).

The final equations in terms of the actual factors for CLI, MAG-CLI, and GO-MAG-CLI and the adsorption capacity for CIP were given by Equations (3)–(5), respectively. Factors variables are denoted as follows: A = *X*_1_, B = *X*_2_, C = *X*_3_, and D = *X*_4_.

Sqrt(q) = 2.08 + 0.5862‧A − 0.3774‧B + 0.1117‧C + 0.0718‧D − 0.2097E[1] − 0.2156E[2] − 0.0521‧AB + 0.0185‧AD + 0.044‧AE[1] − 0.1374‧AE[2] − 0.0339‧BC − 0.1154‧BE[1] − 0.1207‧BE[2] − 0.3838‧CE[1] + 0.3332‧CE[2] − 0.0228‧DE[1] − 0.0279‧DE[2] + 0.0949‧B2 + 0.045‧C2 − 0.0403‧D2 + 0.015‧ABC + 0.0396‧ABE[1] + 0.0256‧ABE[2] − 0.0683‧ACE[1] + 0.1229‧ACE[2] + 0.0031‧ADE[1] − 0.022‧ADE[2] + 0.014‧BCD − 0.1472‧BCE[1] + 0.2274‧BCE[2] + 0.0034‧CDE[1] − 0.0183‧CDE[2] − 0.0807‧A2B − 0.1214‧A2C + 0.3386‧A2E[1] − 0.3314‧A2E[2] + 0.2901‧B2E[1] − 0.1964‧B2E[2] + 0.0682‧C2E[1] − 0.1487‧C2E[2] − 0.0383‧ABCE[1] + 0.0451‧ABCE[2] − 0.1637‧A2B2 + 0.0987‧A2BE[1] − 0.0921‧A2BE[2] + 0.1855‧A2CE[1] − 0.0826‧A2CE[2] − 0.1318‧AB2E[1] + 0.144‧AB2E[2] − 0.007‧ABCDE[1] + 0.0236‧ABCDE[2] − 0.5789‧A2B2E[1] + 0.6417‧A2B2E[2](2)

*Y*^1/2^ = −16.01 + 1.51*X*_1_ + 4.84*X*_2_ + 0.07*X*_3_ + 0.04*X*_4_ − 0.41*X*_1_*X*_2_ − 0.005*X*_1_*X*_3_ + 0.0002*X*_1_*X*_4_ − 0.01*X*_2_*X*_3_ + 0.0001*X*_3_*X*_4_ − 0.02*X*_1_^2^ − 0.34*X*_2_^2^ + 0.001*X*_3_^2^ − 0.002*X*_4_^2^
− 0.0001*X*_1_*X*_2_*X*_3_ + 0.00001*X*_2_*X*_3_*X*_4_ + 0.01*X*_1_^2^*X*_2_ + 0.03*X*_1_*X*_2_^2^ + 0.01*X*_1_^2^*X*_2_
+ 0.03*X*_1_*X*_2_^2^ − 0.0005*X*_1_^2^*X*_2_^2^ (3)

*Y*^1/2^ = 25.38 − 1.59*X*_1_ − 6.98*X*_2_ + 0.03*X*_3_ + 0.05*X*_4_ + 0.46*X*_1_*X*_2_ + 0.002*X*_1_*X*_3_
+ 0.00005*X*_1_*X*_4_ + 0.005*X*_2_*X*_3_ − 0.0002*X*_3_*X*_4_ + 0.03*X*_1_^2^ + 0.46*X*_2_^2^ + 0.003*X*_3_^2^
− 0.002*X*_4_^2^ + 0.0003*X*_1_*X*_2_*X*_3_ + 0.00001*X*_2_*X*_3_*X*_4_ − 0.01*X*_1_^2^*X*_2_ − 0.00005*X*_1_^2^*X*_3_
− 0.03*X*_1_*X*_2_^2^ − 0.000001*X*_1_*X*_2_*X*_4_ + 0.0005*X*_1_^2^*X*_2_^2^ (4)

*Y*^1/2^ = 0.15*X*_1_ + 0.81*X*_2_ − 0.02*X*_3_ + 0.04*X*_4_ − 0.04*X*_1_*X*_2_ + 0.003*X*_1_*X*_3_ + 0.0002*X*_1_*X*_4_
− 0.01*X*_2_*X*_3_ + 0.0001*X*_3_*X*_4_ − 0.002*X*_1_^2^ − 0.05*X*_2_^2^ + 0.001*X*_3_^2^ − 0.002*X*_4_^2^
+ 0.00005*X*_1_*X*_2_*X*_3_ + 0.000004*X*_2_*X*_3_*X*_4_ + 0.001*X*_1_^2^*X*_2_ − 0.0001*X*_2_^2^*X*_3_ + 0.003*X*_1_*X*_2_^2^
+ 0.000001*X*_1_*X*_2_*X*_3_*X*_4_ − 0.0001*X*_1_^2^*X*_2_^2^ (5)

### 3.3. Effects of Variables on CIP Adsorption

**Figure 11:**
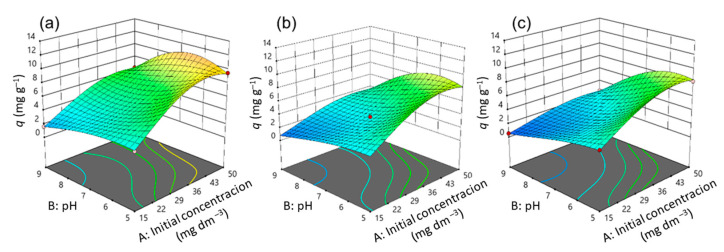
A 3D representation of response surface plots for the CLI adsorbent at a contact time of 20 min for (**a**) 10 °C, (**b**) 17.5 °C, and (**c**) 25 °C.

**Figure 12:**
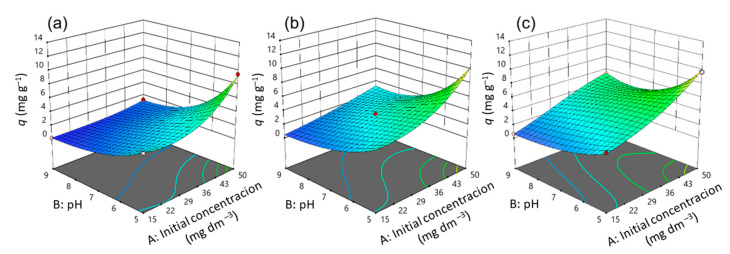
A 3D representation of response surface plots for the MAG-CLI adsorbent at a contact time of 20 min for (**a**) 10 °C, (**b**) 17.5 °C, and (**c**) 25 °C.

**Figure 13:**
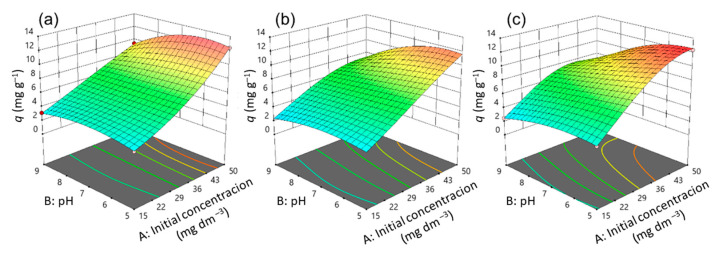
A 3D representation of response surface plots for the GO-MAG-CLI adsorbent at a contact time of 20 min for (**a**) 10 °C, (**b**) 17.5 °C, and (**c**) 25 °C.

Three-dimensional diagrams (Figure 11, Figure 12 and Figure 13) and contours (Figure S4) help to clarify the effects of independent variables on the responses at different points [36]. The optimal conditions that provide the maximum adsorption capacity for CIP using the CLI, MAG-CLI, and GO-MAG-CLI adsorbents are obtained using the multicriteria optimization process and are presented via contour diagrams (Figure S4) and in Table 7.

The effects of the operational parameters including the initial CIP concentration, pH, temperature, and contact time on the adsorption of ciprofloxacin onto CLI, MAG-CLI, and GO-MAG-CLI adsorbents were studied. Figure 11, Figure 12 and Figure 13 show three-dimensional surface plots related to the interactive effects of the initial CIP concentration and pH at three different temperatures and for all three adsorbents at a fixed contact time of 20 min. The contact time is one of the crucial parameters in the determination of wastewater treatment cost-effectiveness. The removal efficiency of contaminants usually increases with the contact time until the equilibrium is reached [66,67]. Accordingly, the adsorption capacity of all three studied CLI-based adsorbents increased with increasing contact time, and the results at the maximum studied time are shown.

The adsorption capacity for CIP was significantly dependent on its initial concentration, and in general the adsorption capacity increased by increasing the initial antibiotic concentration. The enchantment of the adsorption capacity for CIP by increasing the CIP concentration in the solution could be a result of improving the concentration gradient as the adsorption driving force, as well as the availability of the uncovered adsorbent’s surface area at the beginning of the process [19,27]. A comparable result was obtained for CIP removal using activated carbon [67].

The pH value proved to be the second most significant factor for the CIP adsorption onto CLI, MAG-CLI, and GO-MAG-CLI, since the pH controls the nature of the adsorbent’s surface and adsorbate molecules, and in turn the adsorbate–adsorbent interactions [7]. As Figure 11, Figure 12 and Figure 13 indicate, the adsorption capacity of the prepared adsorbents for CIP slightly increases with the pH decreasing from 9 to 5. Consequently, the optimum pH of CIP adsorption onto CLI-based adsorbents is found to be around 5. Since the CIP molecule is a zwitterion, with p*K*a values of 5.90 and 8.89 for the amine and carboxyl group, respectively, it exists in a cationic form at pH levels below 5.90, whereas pH values above 8.89 favor its anionic form [7,8]. Accordingly, at the pH of 5, CIP is mainly present as a cation, and the surfaces of the CLI, MAG-CLI, and GO-MAG-CLI are negatively charged. Therefore, the CLI-based adsorbents’ particle and CIP molecules attracted each other, and the adsorption efficiency increased. On the other hand, the adsorption efficiency was reduced to a pH higher than 8.89 due to the electrostatic repulsion between an anionic form of the CIP molecule and the negatively charged surface of the adsorbents. A similar result was reported by Najafpoor et al. [10] for CIP removal from synthetic wastewaters using *γ*-Al_2_O_3_ nanoparticles.

The interaction of the initial CIP concentration and pH for different temperatures showed that GO-MAG-CLI had a maximum adsorption capacity for CIP at the highest applied temperature (Figure 13c) and CLI had a maximum adsorption capacity at the lowest applied temperature (Figure 11a), while the capacity of MAG-CLI did not change significantly with the temperature (Figure 12). Since the adsorption process mainly occurs as an exothermic process, the decrease in CLI adsorption ability with the temperature increase can be explained by Le Chatelier’s principle. A degree of the decrease in adsorption ability depends on the heat of adsorption; the higher the adsorption heat, the higher the temperature needed to decrease the adsorption ability to some extent [68]. The enhancement in the mobility of CIP molecules with an increase in the solution temperature can, consequently, lead to the equilibrium shift towards desorption. A similar trend was observed for the cephalexin adsorption by Fe_3_O_4_ nanoparticles [27]. The lower adsorption ability of MAG-CLI for CIP in comparison to the ability of pristine CLI could be ascribed to the slightly degraded CLI surface after the coating of MAG nanoparticles, which resulted in a decrease in the number of active sites on the surface available for the CIP adsorption.

As expected, GO-MAG-CLI showed the highest adsorption capacity for CIP at all studied temperatures. It was found that GO itself had a great adsorption ability for CIP removal from water media [34]. A coating of CLI with GO could enlarge the active surface area and subsequently the amount of surface sites available for the antibiotic molecule adsorption [29,69].

### 3.4. Adsorption Isotherm Study

Adsorption isotherms describe the relationships between the amount of absorbed antibiotic molecule onto the adsorbent and the equilibrium antibiotic concentration in the solution. The adsorption of CIP on CLI, MAG-CLI, and GO-MAG-CLI was studied at 10, 15, and 20 °C for the initial CIP concentrations of 15, 25, 32.5, and 50 mg dm^−3^ at pH = 5 and a solid-to-liquid ratio of 1:250, for which the previous study showed the highest CIP removal efficiency [45].

**Figure 14:**
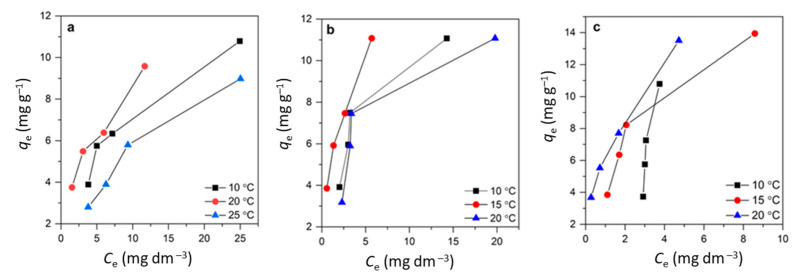
The adsorption isotherms for CIP on (**a**) CLI, (**b**) MAG-CLI, and (**c**) GO-MAG-CLI; *q*_e_ is the amount of the adsorbed CIP (mg per 1 g of the adsorbents) and *C*_e_ is the CIP solution concentration at equilibrium.

The adsorption capacity levels of CLI, MAG-CLI, and GO-MAG-CLI increased with the initial CIP concentration at all studied temperatures (Figure 14). In the case of CLI, the adsorption capacity slightly decreased with temperature, while the capacity of GO-MAG-CLI increased with the temperature increase for all studied initial CIP concentrations. However, an increase in temperature did not cause any significant changes in the adsorption capacity of MAG-CLI, which was found to be 11.07 mg g^−1^ for all investigated temperatures and initial CIP concentrations. This suggests that the MAG coating did not influence the CIP adsorption.

The adsorption equilibrium data given in Figure 14 were further analyzed using commonly applied empirical adsorption isotherm models [66,70]. Among the two parameters models, only the Langmuir and Freundlich isotherm models gave acceptable fits. The adsorption isotherm parameters were determined using a linear regression analysis according to the equations given in Table 8.

The results are summarized in Table 9. As can be seen from the obtained values of the linear regression correlation coefficients (*R*^2^), the Langmuir model gave a slightly better description for the CIP adsorption onto CLI, MAG-CLI, and GO-MAG-CLI. The maximum calculated capacities were 15.15 mg CIP g^−1^, 21.25 mg CIP g^−1^, and 47.91 mg CIP g^−1^ at 15 °C for CLI, MAG-CLI, and GO-MAG-CLI, respectively. Considering the assumptions of the Langmuir model [71], it could be concluded that one active site at the surface of adsorbents is occupied by only one CIP molecule, and that the CIP adsorption could not proceed beyond the monolayer. Furthermore, the Langmuir separation factor *R*_L_ (not shown) was in the range of 0–1, indicating that the adsorption is a favorable process.

### 3.5. Adsorption Kinetics

**Figure 15:**
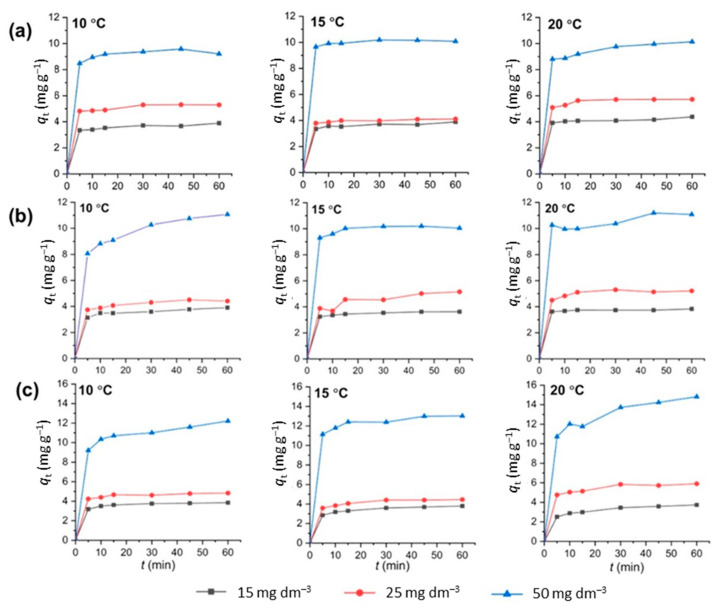
Adsorption kinetics at different temperatures for CIP on (**a**) CLI, (**b**) MAG-CLI, and (**c**) GO-MAG-CLI; *q*_t_ is the amount of the adsorbed CIP (mg CIP g^−1^ CLI) after time *t*.

To investigate the dynamics of the CIP adsorption reaction, i.e., the relation between the adsorption time and adsorption capacity, as well as the process controlling mechanism, kinetic studies were performed [36]. Herein, the CIP adsorption dependence on time was investigated at 10, 15, and 20 °C for CIP solutions with *C*_0_ = 15, 25, and 50 mg dm^−3^. The adsorption capacities of CLI, MAG-CLI, and GO-MAG-CLI for CIP as a function of the contact time are shown in Figure 15.

For all three adsorbents, the CIP uptake increases rather sharply in the first 5 min of the adsorption for all studied temperatures and initial CIP concentrations. Further, the adsorption occurs more slowly. More than 85% of the maximum adsorption capacity is achieved within this first 5 min, which indicates fast adsorption kinetics of CIP onto CLI-based adsorbents.

The experimental data from Figure 15 were analyzed using two reaction-based kinetic models, i.e., Lagergren’s pseudo-first-order and pseudo-second-order kinetic models, and the interparticle diffusion model. Lagergren’s pseudo-first-order model can be expressed as follows:

*dq*_t_/*dt* = *k*_1_(*q*_e_ − *q*_t_) (6)

where *q*_e_ and *q*_t_ are the amounts of adsorbed CIP (mg g^−1^) at equilibrium and at time *t*, respectively; *k*_1_ (min^−1^) is the equilibrium rate constant in the pseudo-first-order model. The Equation (6) can further be modified into a linear form by integration using the boundary conditions (*q*_t_ = 0 − *q*_t_, and *t* = 0 − *t*):

log(*q*_e_ − *q*_t_) = log*q*_e_ − (*k*_1_/2.303)*t* (7)

According to Equation (7), the plot of log(*q*_e_ − *q*_t_) vs. time (*t*) provides estimates of *q*_e_ and *k*_1_ from the intercept and the slope, respectively [72].

The pseudo-second-order equation assumes that the adsorption capacity is directly proportional to the number of occupied active sites on the adsorbent surface. This model has the advantage of studying the adsorption kinetics for low-concentration solutions [66,70]. The differential equation for the pseudo-second-order model can be represented as:

*dq*_t_/*dt* = *k*_2_(*q*_e_ − *q*_t_)^2^ (8)

where *k*_2_ (g mg^−1^ min^−1^) stands for the pseudo-second-order rate constant. By integrating Equation (8) in the same limits as above, the equation can be easily linearized into:

*t*/*q*_t_ = 1/(*k*_2_*q*_e_^2^) + (1/*q*_e_)*t* (9)

The linear plot of *t*/*q*_t_ vs. *t* gives 1/*q*_t_ as the slope and 1/(*k*_2_*q*_e_^2^) as the intercept.

By applying the two reaction-based kinetic models to the experimental data, the correlation coefficient (*R*^2^) values lower than 0.80 (not shown) obtained for the linear relation between log(*q*_e_ − *q*_t_) and *t* indicate that the adsorption of CIP did not follow the pseudo-first-order kinetic model. The adsorption of CIP on CLI-based adsorbents could be better described by the pseudo-second-order kinetic model, since the obtained *R*^2^ values of that model almost reached one. The kinetic parameters for the adsorption of CIP onto CLI, MAG-CLI, and GO-MAG-CLI calculated from the linear plots of the pseudo-second-order kinetic model are given in Table 10. According to the assumption of the pseudo-second-order model, chemisorption is the limiting step that controls the surface adsorption process, and the sites are occupied proportional to the square of the number of vacant surface sites [36]. Additionally, the maximum calculated capacities obtained for the highest studied concentration, 10.34 mg CIP g^−1^ (at 20 °C), 9.70 mg CIP g^−1^ (at 20 °C), and 15.43 mg CIP g^−1^ (at 10 °C) for CLI, MAG-CLI, and GO-MAG-CLI, respectively, were close to the experimentally obtained values. The Lagergren’s pseudo-second-order rate constants (*k*_2_) were in the range of 0.0329–0.5164 g mg^−1^ min^−1^ for all three studied adsorbents. The unclear trend of changing *k*_2_ values with temperature and initial CIP concentration changes indicates the complex mechanism of CIP adsorption onto the CLI-based adsorbents. Comparable results were obtained for the CIP adsorption by clays and *γ*-Al_2_O_3_ nanoparticles [10,14].

The application of the Weber–Morris diffusion model to the experimental data (not shown) resulted in parameter *I* (related to the thickness of the boundary layer) values higher than one, which indicated that the intra-particle diffusion is not the rate-limiting step [35].

## 4. Conclusions

Herein, the magnetic clinoptilolite coated with graphene oxide was synthesized using the combination of the microwave-assisted method and ultrasonication. The non-conventional approach of using microwave irradiation in composite synthesis has been proven to reduce the environmental impact and simplify the procedure. The synthesized composite showed a high adsorption ability towards the antibiotic ciprofloxacin present in the water media. The ciprofloxacin adsorption was optimized using the response surface methodology through the central composite factorial design framework. The highest adsorption capacity was achieved under the optimal conditions of a 48.47 mg dm^−3^ initial CIP concentration and pH of 5.10 at 24.78 °C and with a 19.20 min contact time. The final composite showed a higher adsorption capacity for ciprofloxacin than pristine clinoptilolite. According to the statistical analysis, the considered levels of the initial ciprofloxacin concentration and pH had the most significant effect on the ciprofloxacin adsorption. The process of ciprofloxacin adsorption was well described by the Langmuir adsorption isotherm and Lagergren’s pseudo-second-order kinetic model.

## Figures and Tables

**Table 1:** Design parameters and response variables selected for the experiment.

Factor	Units	Levels		
		−1	0	+1
X_1_: A—Initial CIP concentration	mg dm^−3^	15	32.5	50
X_2_: B—pH		5	7	9
X_3_: C—Temperature	°C	10	17.5	25
X_4_: D—Contact time	min	5	12.5	20
		E[1]		E[2]
X_5_: E—Adsorbent	Categorical	CLI	MAG-CLI	GO-MAG-CLI
RESPONSE				
Adsorption capacity (*q*)	mg g^−1^			

**Table 2:** The phase analysis of MAG and MAG-CLI samples obtained by quantitative XRD analysis using the RIR method.

Phase	Amount (wt.%)
MAG	
Maghemite (*γ*-Fe_2_O_3_)	38.7
Goethite (FeO(OH))	17.0
Magnetite (Fe_3_O_4_)	10.1
Hematite (*α*-Fe_2_O_3_)	8.4
MAG-CLI	
Clinoptilolite (CLI)	69.4
Maghemite (*γ*-Fe_2_O_3_)	23.5

**Table 3:** Mössbauer parameters for the MAG nanoparticles.

Component	*δ*^1^, mm s^−1^	Δ*E*_Q_ ^2^, mm s^−1^	*B*_hf_ ^3^, T	Area, %	Phase
Sextet	0.37	–0.20	51.53	9.5	*α*−Fe_2_O_3_
Sextet	0.37	–0.26	38.01	14.6	*α*−FeOOH
Sextet	0.69 0.25	0.06 –0.04	46.19 48.69	6.6 5.2	Fe_3_O_4_ octahedral Fe_3_O_4_ tetrahedral
Sextet	0.33	0.00	29.71 *	64.0	*γ*−Fe_2_O_3_

^1^ Isomer shift relative to α–Fe; ^2^ quadrupole splitting; ^3^ hyperfine magnetic field.

**Table 4:** Mössbauer parameters for the MAG-CLI sample.

Component	*δ*^1^, mm s^−1^	Δ*E*_Q_ ^2^, mm s^−1^	*B*_hf_ ^3^, T	Area, %	Assignation
Sextet	0.37	–0.03	36.99 *	68.6	*γ*−Fe_2_O_3_
Doublet	0.34	0.66	–	31.4	*γ*−Fe_2_O_3_ superparamagnetic

^1^ Isomer shift relative to α–Fe; ^2^ quadrupole splitting; ^3^ hyperfine magnetic field.

**Table 5:** Textural properties of CLI, GO-CLI, MAG-CLI, and GO-MAG-CLI samples.

Sample	*S*_BET_ ^1^, m^2^ g^−1^	*V*_tot_ ^2^, cm^3^ g^−1^
CLI	24.5	0.099
GO-CLI	37.4	0.120
MAG-CLI	52.1	0.180
GO-MAG-CLI	64.8	0.219

^1^ Specific surface area; ^2^ total pore volume.

**Table 6:** The relative contents of elements (at.%) in the studied samples as determined by the XPS analysis.

Element	CLI	MAG-CLI	GO-MAG-CLI
	at.%		
O	60.6	60.6	48.8
C	4.7	18.5	27.2
Fe	–	5.7	4.2
Si	26.8	18.5	16.3
Al	5.4	3.9	2.2
Ca	1.8	1.6	0.9
K	0.9	0.6	0.4

**Table 7:** Optimal conditions for the CIP adsorption onto the prepared CLI-based adsorbents.

Adsorbent	Optimal Solution
CLI	*C*_0_(CIP) = 50 mg dm^−3^ pH = 6.41 *T* = 9.85 °C *t* = 18.95 min
MAG-CLI	*C*_0_(CIP) = 50 mg dm^−3^ pH = 5 *T* = 20.98 °C *t* = 13.70 min
GO-MAG-CLI	*C*_0_(CIP) = 48.47 mg dm^−3^ pH = 5.10 *T* = 24.78 °C *t* = 19.20 min

**Table 8:** Equations of the used two-parameter adsorption isotherm models [71].

Isotherm Model	Equation *	Model Parameters
Langmuir	1/*q*_e_ = [1/(*Q*_max_*b*_L_)] × 1/*C*_e_ + 1/*Q*_max_	*Q*_max_, *b*_L_
Freundlich	log*q*_e_ = log*K*_F_ + (1/*n*) log*C*_e_	*K*_F_, *n*

Note: * *q*_e_ is the amount of CIP adsorbed at equilibrium (mg g^−1^); *C*_e_ is the equilibrium liquid-phase concentration (mg dm^−3^). In the Langmuir isotherm model, *Q*_max_ (mg g^−1^) and *b*_L_ (dm^3^ mg^−1^) are the Langmuir constants related to the maximum adsorption capacity and adsorption energy, respectively. In Freundlich isotherm model, *K*_F_ (mg g^−1^(dm^3^ mg^−1^)^1/*n*^) and n are isotherm parameters characterizing the adsorption capacity and intensity, respectively.

**Table 9:** The parameters obtained by the applied adsorption isotherm models for the adsorption of CIP onto CLI, MAG-CLI, and GO-MAG-CLI; *R*^2^ is the correlation coefficient.

		Langmuir Isotherm Model			Freundlich Isotherm Model		
	T, °C	*Q*_max_, mg g^−1^	*b*_L_, dm^3^ mg^−1^	*R* ^2^	*K*_F_, mg g^−1^(dm^3^ mg^−1^)^1/n^	*n*	*R* ^2^
CLI	10	15.15	0.14	0.9985	2.42	2.40	0.9838
	15	11.97	0.11	0.9999	2.28	1.92	0.9829
	20	12.60	0.23	0.9990	3.30	2.57	0.9981
MAG-CLI	10	14.91	0.15	0.9764	2.52	1.86	0.9679
	15	21.25	0.05	0.9813	1.44	1.51	0.9792
	20	21.00	0.08	0.9922	2.05	1.58	0.9967
GO-MAG-CLI	10	17.43	0.12	0.9779	2.28	1.61	0.9148
	15	47.91	0.02	0.9781	1.47	1.30	0.9707
	20	41.78	0.02	0.9420	1.32	1.36	0.9192

**Table 10:** Rate constants for Lagergren’s pseudo-second-order kinetic model for the adsorption of CIP on CLI, MAG-CLI, and GO-MAG-CLI (*R*^2^ is the correlation coefficient of the linear regression).

		Lagergren’s Pseudo-Second-Order Rate Parameters								
		CLI			MAG-CLI			GO-MAG-CLI		
*C*_0_, mg dm^−3^	*T*, °C	*k*_2_ ^1^, g mg^−1^ min^−1^	*q*_e_ ^2^, mg g^−1^	*R* ^2^	*k*_2_, g mg^−1^ min^−1^	*q*_e_, mg g^−1^	*R* ^2^	*k*_2_, g mg^−1^ min^−1^	*q*_e_, mg g^−1^	*R* ^2^
15	10	0.1751	3.91	0.9992	0.3133	3.43	0.9995	0.0678	3.92	0.9994
	15	0.2079	3.89	0.9992	0.2445	2.86	0.9998	0.1091	3.91	0.9998
	20	0.2006	4.37	0.9991	0.3243	4.26	0.9999	0.2102	3.91	0.9999
25	10	0.1898	5.40	0.9999	0.1159	5.59	0.9999	0.0875	6.06	0.9996
	15	0.3511	4.14	0.9999	0.1310	5.08	0.9991	0.1317	4.58	0.9999
	20	0.2527	5.80	0.9999	0.1258	5.41	0.9969	0.1977	4.89	0.9998
50	10	0.3390	9.40	0.9995	0.1359	8.60	0.9999	0.0196	15.43	0.9991
	15	0.5164	10.15	0.9999	0.0861	7.84	0.9998	0.0620	13.25	0.9998
	20	0.0644	10.34	0.9998	0.0673	9.70	0.9979	0.0329	12.45	0.9989

^1^ Pseudo-second-order rate constant; ^2^ adsorption capacity.

## Data Availability

The data presented in this study are available upon reasonable request from the corresponding author.

## Supplementary Materials

The following supporting information can be downloaded at: https://www.mdpi.com/article/10.3390/nano13040740/s1, Figure S1: Diffraction pattern of MAG sample. Figure S2: Diffraction pattern of MAG-CLI sample. Figure S3: XPS survey spectra from the surfaces of (a) CLI, (b) MAG-CLI, and (c) GO-MAG-CLI. Figure S4: Contour diagrams for (a) CLI, (b) MAG-CLI, and (c) GO-MAG-CLI adsorption capacity optimization. Table S1: The ANOVA results of the model.
