# Supplementary material for: Optimization of Ciprofloxacin Adsorption on Clinoptilolite-Based Adsorbents Using Response Surface Methodology
Source: Nanomaterials (Basel). 2023 Feb 15;13(4):740. doi: 10.3390/nano13040740 (PMC9966051; doi:10.3390/nano13040740)
Supplement: Supplementary file 1 [file nanomaterials-13-00740-s001.zip › nanomaterials-2213420-supplementary.pdf]

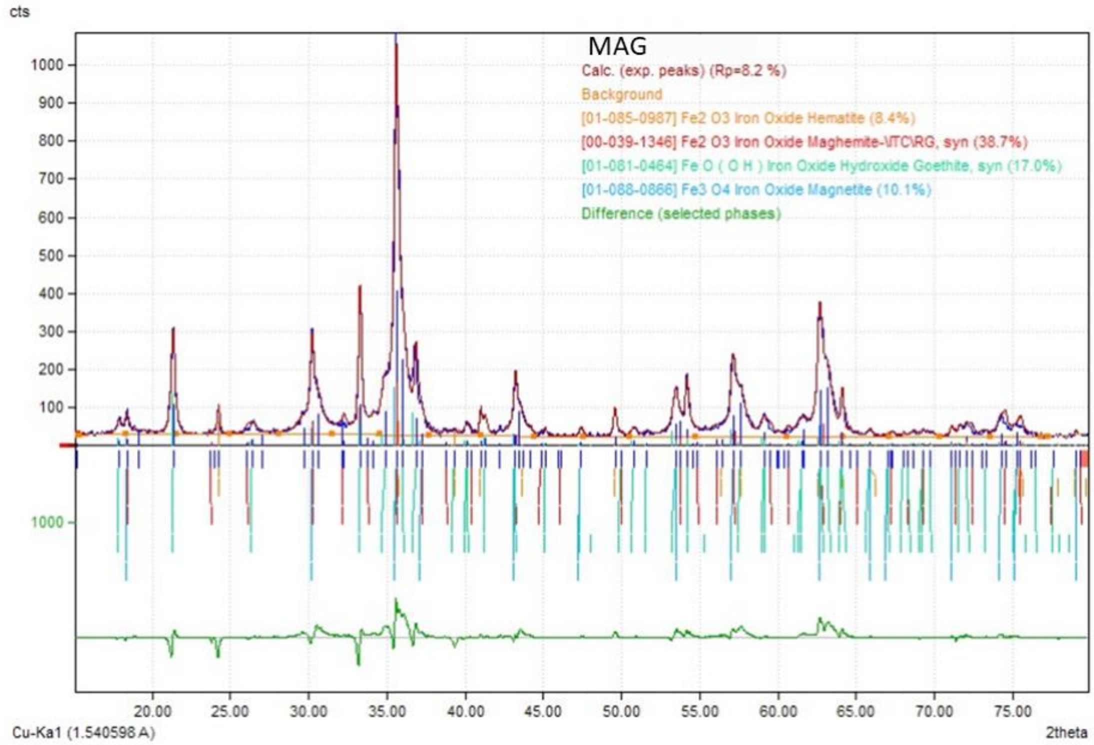


**Figure S1.** Diffraction pattern of MAG sample.

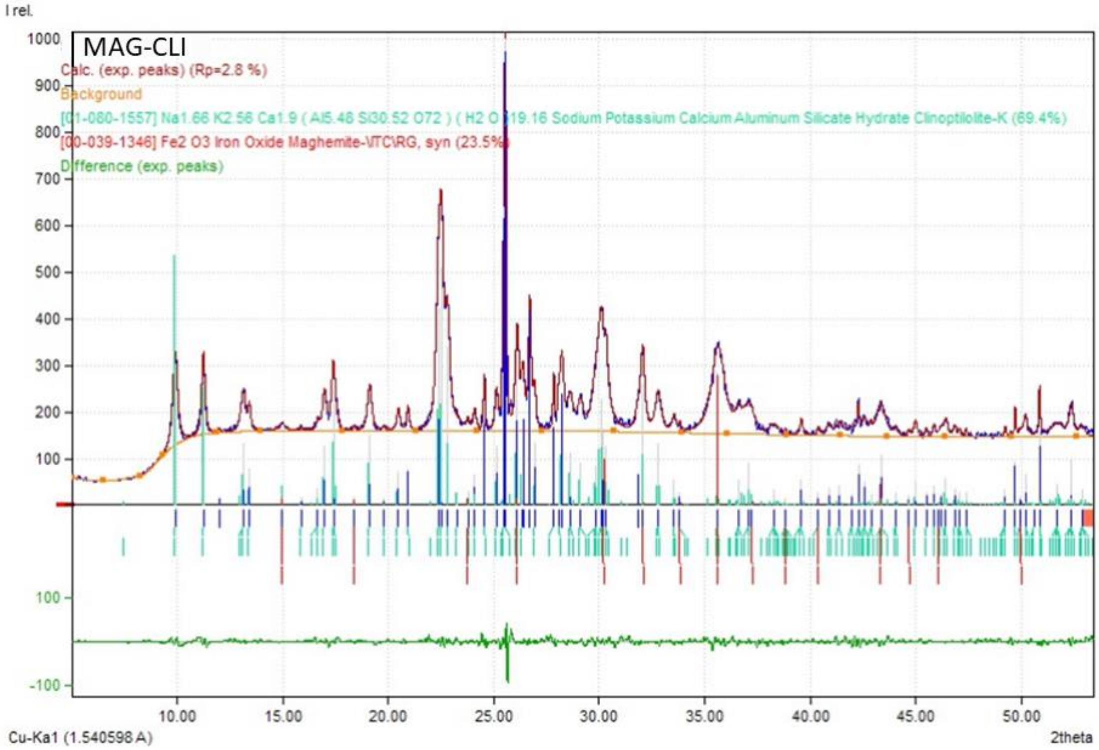


**Figure S2.** Diffraction pattern of MAG-CLI sample.

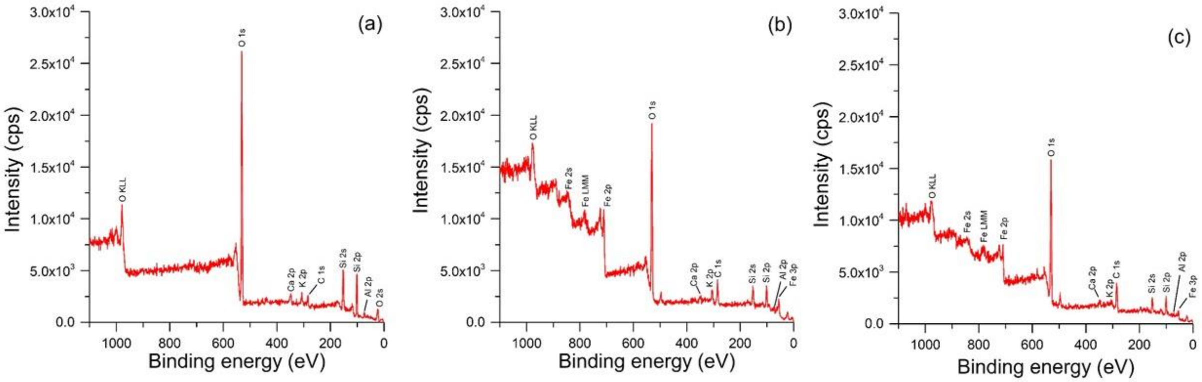


**Figure S3.** XPS survey spectra from the surface of (a) CLI, (b) MAG-CLI and (c) GO-MAG-CLI.

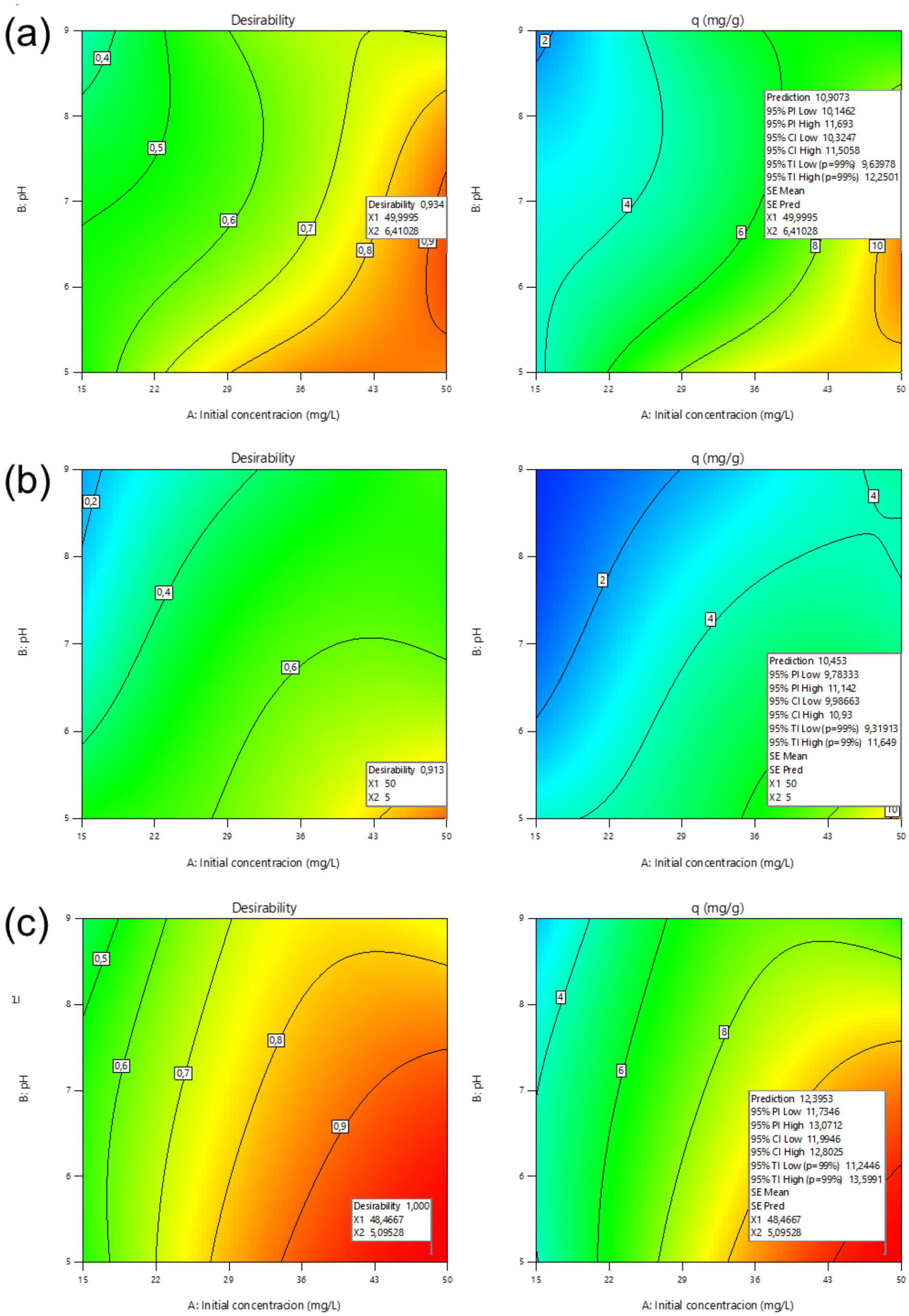


**Figure S4.** Contour diagrams for (a) CLI, (b) MAG-CLI, and (c) GO-MAG-CLI adsorption capacity optimization.

**Table S1.** The ANOVA results of the model.

| Source | Sum of squares | df | Mean square | F-value | *p*-value | Importance |
|---|---|---|---|---|---|---|
| **Model** | 43.95 | 53 | 0.8293 | 596.73 | < 0.0001 | significant |
| A-Initial concentration | 18.56 | 1 | 18.56 | 13351.50 | < 0.0001 | |
| B-pH | 10.89 | 1 | 10.89 | 7836.64 | < 0.0001 | |
| C-Temperature | 0.0007 | 1 | 0.0007 | 0.5325 | 0.4703 | |
| D-Contact time | 0.2781 | 1 | 0.2781 | 200.10 | < 0.0001 | |
| E-Adsorbent | 6.97 | 2 | 3.48 | 2506.08 | < 0.0001 | |
| AB | 0.1302 | 1 | 0.1302 | 93.72 | < 0.0001 | |
| AD | 0.0165 | 1 | 0.0165 | 11.84 | 0.0015 | |
| AE | 0.2206 | 2 | 0.1103 | 79.37 | < 0.0001 | |
| BC | 0.0553 | 1 | 0.0553 | 39.78 | < 0.0001 | |
| BE | 1.71 | 2 | 0.8528 | 613.63 | < 0.0001 | |
| CE | 2.11 | 2 | 1.05 | 758.14 | < 0.0001 | |
| DE | 0.0698 | 2 | 0.0349 | 25.10 | < 0.0001 | |
| $B^2$ | 0.0107 | 1 | 0.0107 | 7.67 | 0.0088 | |
| $C^2$ | 0.0097 | 1 | 0.0097 | 7.00 | 0.0120 | |
| $D^2$ | 0.0078 | 1 | 0.0078 | 5.62 | 0.0233 | |
| ABC | 0.0108 | 1 | 0.0108 | 7.74 | 0.0085 | |
| ABE | 0.1039 | 2 | 0.0519 | 37.38 | < 0.0001 | |
| ACE | 0.3642 | 2 | 0.1821 | 131.04 | < 0.0001 | |
| ADE | 0.0137 | 2 | 0.0068 | 4.92 | 0.0129 | |
| BCD | 0.0094 | 1 | 0.0094 | 6.80 | 0.0132 | |
| BCE | 1.28 | 2 | 0.6383 | 459.31 | < 0.0001 | |
| CDE | 0.0091 | 2 | 0.0045 | 3.27 | 0.0494 | |
| $A^2B$ | 0.0347 | 1 | 0.0347 | 24.97 | < 0.0001 | |
| $A^2C$ | 0.0787 | 1 | 0.0787 | 56.60 | < 0.0001 | |
| $A^2E$ | 0.0765 | 2 | 0.0382 | 27.51 | < 0.0001 | |
| $B^2E$ | 0.0603 | 2 | 0.0302 | 21.70 | < 0.0001 | |
| $C^2E$ | 0.0532 | 2 | 0.0266 | 19.14 | < 0.0001 | |
| ABCE | 0.0567 | 2 | 0.0283 | 20.39 | < 0.0001 | |
| $A^2B^2$ | 0.0390 | 1 | 0.0390 | 28.06 | < 0.0001 | |
| $A^2BE$ | 0.0325 | 2 | 0.0162 | 11.68 | 0.0001 | |
| $A^2CE$ | 0.0921 | 2 | 0.0461 | 33.15 | < 0.0001 | |
| $AB^2E$ | 0.0680 | 2 | 0.0340 | 24.48 | < 0.0001 | |
| ABCDE | 0.0141 | 2 | 0.0071 | 5.09 | 0.0113 | |
| $A^2B^2E$ | 0.3640 | 2 | 0.1820 | 130.97 | < 0.0001 | |
| **Residual** | 0.0500 | 36 | 0.0014 | | | |
| Lack of Fit | 0.0297 | 21 | 0.0014 | 1.04 | 0.4770 | not significant |
| Pure Error | 0.0203 | 15 | 0.0014 | | | |
| **Cor Total** | 44.00 | 89 | | | | |
